# Evaluation of nurses’ knowledge and practice regarding sepsis management: “A case study of adult ICU/HDU setting at CHUK”

**DOI:** 10.1186/s12912-025-03936-7

**Published:** 2025-10-14

**Authors:** Sophie Mukantwari, Faustine Ingabire, Aline Maniragena, Nadine Mukamuvunyi, Kingsley Tobi, Immaculée Barindikije, Carine Higo, Emmanuel Bikorimana, Joseph Mucumbitsi

**Affiliations:** 1 Adult ICU/HDU Department, University Teaching Hospital of Kigali (CHUK), Kigali, Rwanda; 2 Department of Nursing, College of Medicine and Health Sciences, University of Rwanda, Kigali, Rwanda; 3 Department of Microbiology, Adventist School of Medicine (ASOME), Adventist University of Central Africa, Kigali, Rwanda

**Keywords:** Evaluation, Nurse’s knowledge, Practice and sepsis, Septic shock

## Abstract

**Background:**

Sepsis is a life-threatening condition that leads to high mortality in ICU and HDU settings globally. It results from a dysregulated immune response to infection, causing organ failure. Nurses play a key role in early detection and effective management due to their continuous patient care. Despite international guidelines like the Surviving Sepsis Campaign, gaps in knowledge and practice remain, particularly in low-resource settings. This study assessed nurses’ knowledge and practices at CHUK to identify areas for improvement.

**Methodology:**

A quantitative research approach was used with a cross-sectional design. The study population consisted of nurses working in ICU/HDU unit at CHUK, with a sample size covering 51 participants. Descriptive statistics was applied to each variable, and data was presented using tables and frequency distributions. Mean scores and standard deviations were calculated for quantitative variables. Ordinal logistic regression was carried out to study factors associated with knowledge towards knowledge and management of sepsis. Variables with a p-value < 0.05 from the ordinal logistic regression was considered statistically significant.

**Results:**

Among participants, 11.8% had low level of knowledge,58.8% has moderate knowledge and 29.4% had good level of knowledge towards sepsis. Among them 39.2% showed low level of practice, 29.4% showed moderate level of practice and 31.4% good practice. The results from ordinal regression showed that level of education is a factor of knowledge towards sepsis. Those with Bachelor’s degree showed to have inadequate knowledge (P-value = 0.032). The results from ordinal regression showed that training is a factor to practice towards the management of sepsis. Those who were not trained showed inadequate practice (P-value = 0.002).

**Conclusion:**

The study found that nurses had moderate knowledge but poor practice in sepsis management. Lower education levels and lack of sepsis-specific training were key contributing factors. This highlights the need for continuous education and targeted training programs. Future research should explore the lived experiences and challenges nurses face in managing sepsis to inform more effective interventions.

**Supplementary Information:**

The online version contains supplementary material available at 10.1186/s12912-025-03936-7.

## Introduction

Sepsis is a major cause of death in ICU and HDU settings, requiring timely recognition and intervention, particularly by nurses who play a fundamental role in prompt management [1]. Despite global efforts such as the Surviving Sepsis Campaign, gaps in nurses’ knowledge and practice remain prevalent, especially in low-resource settings [2]. At CHUK, where sepsis is frequently encountered, no comprehensive studies have yet assessed nurses’ knowledge and practice concerning sepsis. This study addresses that gap by evaluating both knowledge and practice, provided that essential baseline data to guide targeted clinical training.

Sepsis is a serious global health emergency and one of the leading causes of death worldwide. It poses a particularly high burden in low- and middle-income countries due to limited resources for timely treatment [3] More than 30,000 admissions to ICU due to sepsis in the UK each year, and the number is rising. Mortality rates remain high and there are more deaths in the UK from sepsis than from either breast or colon cancer [4]. Sepsis remains a critical health challenge in India, contributing to 60–80% of annual deaths from infections, with a high ICU prevalence of 56.4%. Common sources include pneumonia, urinary tract infections, and bloodstream infections, predominantly caused by Gram-negative pathogens [5].

Sepsis is a major cause of illness and death among children, affecting an estimated 3 million neonates and 1.2 million children annually. In 2017, the global incidence reached 4.9 million in children under 5 and 23.7 million in those aged 5–19, with 2.9%–8.2% of pediatric ICU admissions involving sepsis [6]. Studies across African countries highlight the high burden and mortality of sepsis. In South Africa, 21% of surgical admissions had sepsis, while Cameroon reported 33% with sepsis and 21% with septic shock. Mortality rates vary widely, from 7% in Gabon to as high as 83% for septic shock in Rwandan ICUs, indicating a critical need for improved sepsis management across the continent [7]. In Rwandan ICUs, 42% of patients were diagnosed with sepsis, 33% with severe sepsis, and 21% with septic shock shortly after admission. These findings underscore the urgent need to assess nurses’ knowledge and management strategies to effectively address the high sepsis burden in critical care [8]. The burden is highest in low- and middle-income countries, particularly in Africa, accounting for around 17 million cases and 3.5 million deaths, with the most affected being newborns, pregnant women, and the elderly [7].

In Rwanda, sepsis poses a major health challenge, largely due to limited healthcare resources that delay its timely recognition and management. A retrospective study in two referral hospitals reported sepsis-related mortality rates of 31.5% without shock and up to 82.9% with septic shock [8]. At CHUK, Rwanda’s principal referral hospital, nurses are essential in the early identification of hospital-acquired sepsis, due to their continuous bedside care, particularly within the adult ICU/ HDU units where sepsis remains a leading cause of death. This original study discovers significant gaps in the knowledge and practices of ICU/ HDU nurses concerning sepsis management. The results are intended to guide clinical training programs, shape healthcare policies, and ultimately contribute to lowering preventable deaths from sepsis.

### Research questions

What is the current level of knowledge among nurses regarding the identification and management of sepsis in critically ill patients admitted to the ICU/HDU at CHUK?

How competent practice are nurses in applying sepsis management protocols in the ICU/HDU setting at CHUK?

What are the major gaps in knowledge and specific training requirements for ICU/HDU nurses at CHUK to optimize the recognition and management of sepsis?

## Methods

### Study design

This study employed a quantitative cross-sectional design, which allowed for the collection of data at a single point in time to assess nurses’ knowledge and practices related to sepsis management.

Cross-sectional designs are commonly used in healthcare research to evaluate current conditions or behaviors across a defined population [9]. Similar studies assessing knowledge and practices in critical care settings have used this design effectively [10].

### Study setting

University Teaching Hospital of Kigali. started in 1928 by the Catholic Church at level of health post/dispensary. Centre Hospitalier Universities de Kigali is one of two University teaching hospitals in Rwanda. CHUK is located in Capital city of Rwanda approximately at 2 km far from commercial zone. It is located in Nyarugenge District. CHUK Hospital has a capacity of 438 beds with an occupancy rate from 91%. It serves a population/hospitalization of more than 9,709/month but it covers also big population/Out Patient Department 14,400 per month (approxim). CHUK hospital has various departments where Infection Prevention Control unit is among in addition to that, IPC Unit is committed to raise knowledge and skills of all staff such that Hospital Acquired infections are reduced and prevented.

The various of specialized departments, including Accident and Emergency, Surgical Ward, Pediatrics, Orthopedics, Internal Medicine, Neurosurgery, Gynecology and Obstetrics, Outpatient Services, Operating Theatre, and Intensive Care Units, among others. Notably, CHUK distinguishes itself from other referral hospitals by receiving a significant number of patients referred from other facilities or directly from accident scenes, many of whom may require extended hospitalization lasting several weeks. It is within this context that CHUK was chosen as the site for this study. Thus, this study is intended to monitor knowledge, skills of nurses working in adult critical care unit and high dependent unit.

### Study population

The study was conducted among nurses working in the Adult Intensive Care Unit (ICU) and High-Dependency Unit (HDU) at the Centre Hospitalier Universitaire de Kigali (CHUK). Nurses with at least six months of working experience in these units were eligible to participate. The entire staff of 58 nurses working in ICU/HDU at CHUK was included in the study, where critically ill patients typically experience extended hospital stays. However, those who were absent due to annual or maternity leave, as well as individuals who declined to take part during the data collection period, were excluded from the study.

### Sampling approach and sample size

To calculate the sample size (n) for estimating a population proportion with a specified margin of error and confidence level, we used Cochran’s formula [11]

$$\:\varvec{n}\varvec{{\prime\:}}=\frac{({\varvec{z}}^{2}\times\:\varvec{p}\times\:\varvec{q})}{{\varvec{e}}^{2}}$$

Z is the Z-value corresponding to the desired confidence level (e.g., 1.96 for a 95% confidence level).

e is the margin of error; p is the estimated proportion of the population = 50%.

q is 1 minus the estimated proportion (1 - q)

$$\bf{\:\varvec{n}\varvec{{\prime\:}}=\frac{\left(1.962\times\:0.50\times\:0.50\right)}{{0.05}^{2}}=384.16\approx\:385}$$

The required sample size for the survey was approximately 385 participants. To account for a potential 10% non-response rate, 10% of this sample size was calculated as follows:

10% of 385 = 0.10 × 385 = 38.5, which was rounded to 39.

Therefore, **the final required sample size was approximately 385 + 39 = 424.**

Since the sample size needed to be a whole number, it was rounded up to 424.

However, due to the limited number of ICU/HDU nurses at CHUK, only 58 were included.

**The formula for sampling from limited population was used** due to the limited number of ICU/HDU nurses at CHUK, only 58 were included. As recommended, future studies should expand to include participants from additional hospitals to improve generalizability.

### Sample size

Adjusting the sample size when **sampling from a small population**

$$\:\varvec{n}=\frac{\varvec{N}\times\:{\varvec{n}}^{\varvec{{\prime\:}}}}{\mathbf{N}+\mathbf{n}\mathbf{{\prime\:}}}$$

Where:

n = corrected sample size; n=?

n’ = sample size for the large population (as calculated from previously given formula); n’= 424.

N = Total population size; *N* = 58 an estimation within one month.

$$\bf{\:\varvec{n}=\frac{58\times\:424}{58+424}=51}\:\bf{\varvec{p}\varvec{a}\varvec{r}\varvec{t}\varvec{i}\varvec{c}\varvec{i}\varvec{p}\varvec{a}\varvec{n}\varvec{t}\varvec{s}}$$

### Sampling strategy

This research used systematic random sampling to select 51 participants from a population of 58 nurses. The sampling interval “K” was calculated as follows:

K = Total population / Sample size = 58 / 51 = 1.13, which was approximated to 1.

Since the required sample was 51 out of 58 nurses, the sampling interval was set at 1, meaning that every nurse on the list was selected until the target of 51 participants was reached.

### Data collection instrument

The study employed a structured questionnaire adapted from prior research by Nakiganda et al., Manika et al. and Almutairi et al. [10, 12, 13].

### Objectives and tool items

The study objectives were addressed through a structured self-administered questionnaire consisting of three main sections.

The first objective, which aimed to assess the sociodemographic characteristics of nurses working in ICU and HDU settings, was addressed in Section I of the tool. This section included 7 items that gathered information on participants’ age, gender, education level, years of experience, unit of work, prior training related to sepsis, and employment status.

Section II: This section comprised 7 statements that assessed understanding of sepsis definitions, causes, early signs and symptoms, diagnostic criteria, complications, and general management principles.

The third objective focused on identifying the current practices employed by nurses in managing sepsis. Section III included 10 statements assessing clinical practice such as early recognition of sepsis, timely initiation of treatment protocols, monitoring vital signs, use of sepsis bundles, effective documentation, communication with medical teams, and ongoing evaluation of patient progress.

### Scoring and cut-offs

The respondents’ knowledge and practice levels were categorized based on predefined percentage thresholds. A composite **s**core of less than 50% was considered to reflect poor knowledge or practice, while scores ranging from 50% to 75% indicated a moderate level. Respondents who scored above 75% were categorized as having excellent knowledge or practice [10, 12, 13].

The revised instrument was then reviewed by a panel of experts, including experienced critical care nurses, a senior ICU physician with extensive experience in sepsis management, and academic professionals. Following this rigorous validation process, the questionnaire achieved a Content Validity Index (CVI) of 0.86, reflecting a high level of content validity.

### Language and cultural adaptation

The tool was adapted and validated for relevance to the Rwandan Context. Minor modifications were made to align with local protocols, and it was reviewed by experts for face and content validity. The questionnaire was first in English (the official medical language in Rwanda) and was not translated, as all participating nurses were proficient. A pilot test was conducted with 5 nurses (excluded from final analysis) to refine clarity and appropriateness.

### Reliability

A Cronbach’s alpha has a value between 0 and 1, where a higher value denotes greater dependability and a lower value, less reliability. Values above 0.7 are generally seen as satisfactory, indicating that all of the instrument’s items are dependable, whereas values below 0.50 are often regarded as unsuitable. The instrument’s Cronbach’s alpha in the current study was higher than 0.7, indicating a high level of internal consistency and reliability for the measurement scale used in the study. Demonstrating that it evaluates nurses’ knowledge and practice on sepsis is an effective [14].

### Ethical approval

The research was conducted in full compliance with the ethical principles outlined in the Declaration of Helsinki. Ethical approval was obtained from the Institutional Review Board of Kigali University Teaching Hospital (CHUK), ensuring that the study respected participant autonomy, confidentiality, and welfare. Informed consent was obtained from all participants, who were made aware that participation was voluntary and that they could withdraw at any time without penalty. Confidentiality and data protection measures were rigorously applied in accordance with ethical standards.

### Questionnaire administration

Participants filled out a self-administered questionnaire that had been given to them by the researcher. They answered the questions on their own, and it was estimated that completing the questionnaire would take about 15 min. The researcher handed out the questionnaires to eligible participants after their regular staff meeting and approximately one hour before the end of their shift. A designated drop box was provided for submitting the completed questionnaires, which were then collected daily by the research team.

### Data analysis

Data analysis was conducted utilizing Statistical Package for Social Sciences (SPSS) version 25.

Descriptive statistics was applied to each variable, and data was presented using tables and frequency distributions. Mean scores and standard deviations were calculated for quantitative variables. Ordinal logistic regression was carried out to study factors associated with knowledge towards knowledge and management of sepsis. Variables with a p-value < 0.05 from the ordinal logistic regression was considered statistically significant.

### Data management

All completed qes were securely stored in locked cabinets, while electronic data were saved on a password-protected computer accessible only to the principal investigator. Confidentiality was ensured through strict data protection measures, with access limited to authorized personnel and all sensitive documents kept in a secured area.

## Results

The study assessed 51 ICU/HDU nurses at CHUK with diverse demographic characteristics. Gender distribution was nearly equal, with 52.9% female and 47.1% male participants. The majority (54.9%) were aged between 30 and 39 years, indicating a predominantly young and active workforce, while smaller proportions were aged 40–49 (21.6%), 20–29 (17.6%), and 50+ (5.9%). Most nurses were married (68.6%), which may reflect stability in personal life that could positively influence their professional roles. In terms of education, over half (54.9%) held a Bachelor’s degree, 37.3% had a Diploma, and only 7.8% possessed a Master’s degree, suggesting a workforce with basic to intermediate qualifications. Regarding ICU/HDU experience, 33.3% had 4–6 years, 31.4% had less than one year, while 17.6% each had 1–3 years and 7 + years of experience, showing a mix of novice and seasoned staff. Notably, only 21.6% reported having received sepsis-specific training, highlighting a substantial training gap that could impact care quality and patient outcomes in sepsis management (Table 1).

**Table 1:** Demographic information of participants

Variables		*n*	%
Gender of participants	Female	27	52.9
	male	24	47.1
Age of participants	20–29 years old	9	17.6
	30–39 years old	28	54.9
	40–49 years old	11	21.6
	50 and above	3	5.9
Marital status	Married	35	68.6
	Single	15	29.4
	Widow	1	2.0
Highest education level obtained	Diploma in Nursing	19	37.3
	Bachelors’ degree in Nursing	28	54.9
	Master’s degree in Nursing	4	7.8
Year of experience in ICU/HDU	Less than 1 year	16	31.4
	1–3 years	9	17.6
	4–6 years	17	33.3
	7 years and above	9	17.6
Evert received specific training in sepsis management	Yes	11	21.6
	No	40	78.4

Generally, 69.7 of participants answered correctly the questions concerning knowledge about sepsis. The mean score of knowledge about sepsis among participants was 69 with a standard deviation of 38. The highest score was on definition of sepsis with a mean score of 98 with a standard deviation of 14 followed by answering correctly to the role invasive devices play in increasing the risk of sepsis in critically ill patients with a mean score of 92 with a standard deviation of 27 The lowest score was on common signs of sepsis with a mean score of 35 with a standard deviation of 48 (Table 2).

**Table 2:** Scores on knowledge towards sepsis

Variables	Response	*n*	%	Mean	STDEV
What is sepsis	Right	50	98	98	14
	Wrong	1	2		
Which microorganisms are most commonly responsible for sepsis?	Right	21	41	41	49
	Wrong	30	38.8		
Which of the following is a common sign of sepsis?	Right	18	35.3	35	48
	Wrong	33	64.7		
How do underlying conditions such as diabetes, immunosuppression, aseptic wounds, and chronic kidney disease contribute to the development of sepsis in ICU settings?	Right	39	76.5	76	42
	Wrong	12	23.5		
According to the surviving sepsis campaign guidelines, what is the recommended time frame for administering antibiotics after sepsis is suspected?	Right	39	76.5	76	42
	Wrong	12	23.5		
What role do invasive devices play in increasing the risk of sepsis in critically ill patients	Right	47	92.2	92	27
	Wrong	4	7.8		
What is the initial fluid resuscitation recommendation for a patient with septic shock?	Right	35	68.6	68	46
	Wrong	16	31.4		

Most participants demonstrated a moderate to high level of knowledge on sepsis-related topics (Table 2).

**Fig. 1:**
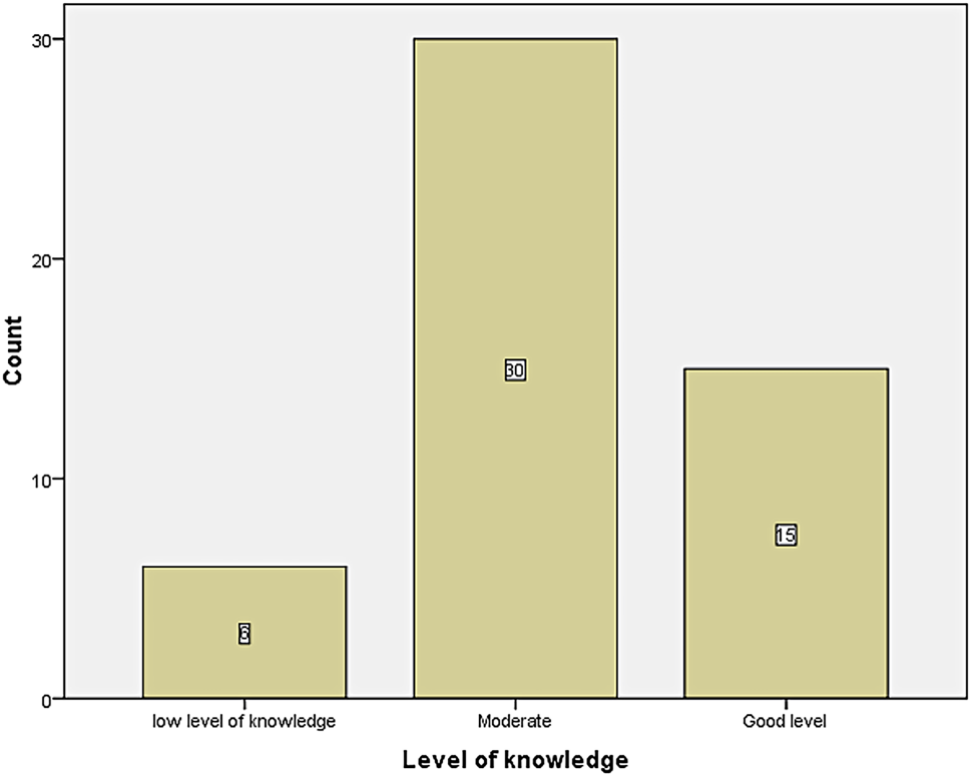
Nurses’ knowledge level regarding sepsis

As shown in Fig. 1, the majority of nurses demonstrated a moderate level of knowledge regarding sepsis management. The level of knowledge was identified, the majority 30 (58%) has moderate knowledge. Those who have good level of knowledge are 15(29.4%).

Generally, 62.9 of participants reported the positive management of sepsis. With a mean score of 62 and a standard deviation of 45.

The highest score was on the best first line treatment for sepsis with a mean score of 96 with a standard deviation of 19, followed by familiarity with Sepsis Six care bundles with a mean score of 88 and a standard deviation of 32. The lowest score was on resources utilized for information on sepsis management and training /educational programs focused on sepsis management in the past years with a mean score of 29 and a standard deviation of 46 for each (Table 3).

**Table 3:** Nurses’ practices towards sepsis management

Variables	Response	*n*	%	Mean	STDEV
How often do you assess the risk of sepsis in ICU/HDU patients?	Right	20	39.2	39	49
	Wrong	31	60.8		
Which of the following is the best first line treatment for sepsis?	Right	49	96.1	96	19
	Wrong	2	3.9		
What laboratory tests are essential for diagnosing sepsis?	Right	21	41.2	41	49
	Wrong	30	58.8		
How familiar are you with the Sepsis Six care bundle (oxygen therapy, blood culture, antibiotics, fluids, lactate, urine output)?	Right	45	88.2	88	32
	Wrong	6	11.8		
How do early identification and timely intervention impact the prognosis of sepsis in ICU patients?	Right	42	82.4	82	38
	Wrong	9	17.6		
What are the key factors that contribute to the rapid progression of sepsis in ICU patients?	Right	45	88.2	88	32
	Wrong	6	11.8		
What resources do you utilize for information on sepsis management (select all that apply)	Right	15	29.4	29	46
	Wrong	36	70.6		
How do organ dysfunction and sepsis-induced immunosuppression interact in critically ill ICU patients?	Right	39	76.5	76	42
	Wrong	12	23.5		
Have participated in any training or educational programs Focused on sepsis management in the past years?	Yes	15	29.4	29	46
	No	36	70.6		
Do you believe enhanced knowledge and competency would improve patient’s outcomes in sepsis management?	Right	30	58.8	58	49
	Wrong	21	41.1		

**Fig. 2:**
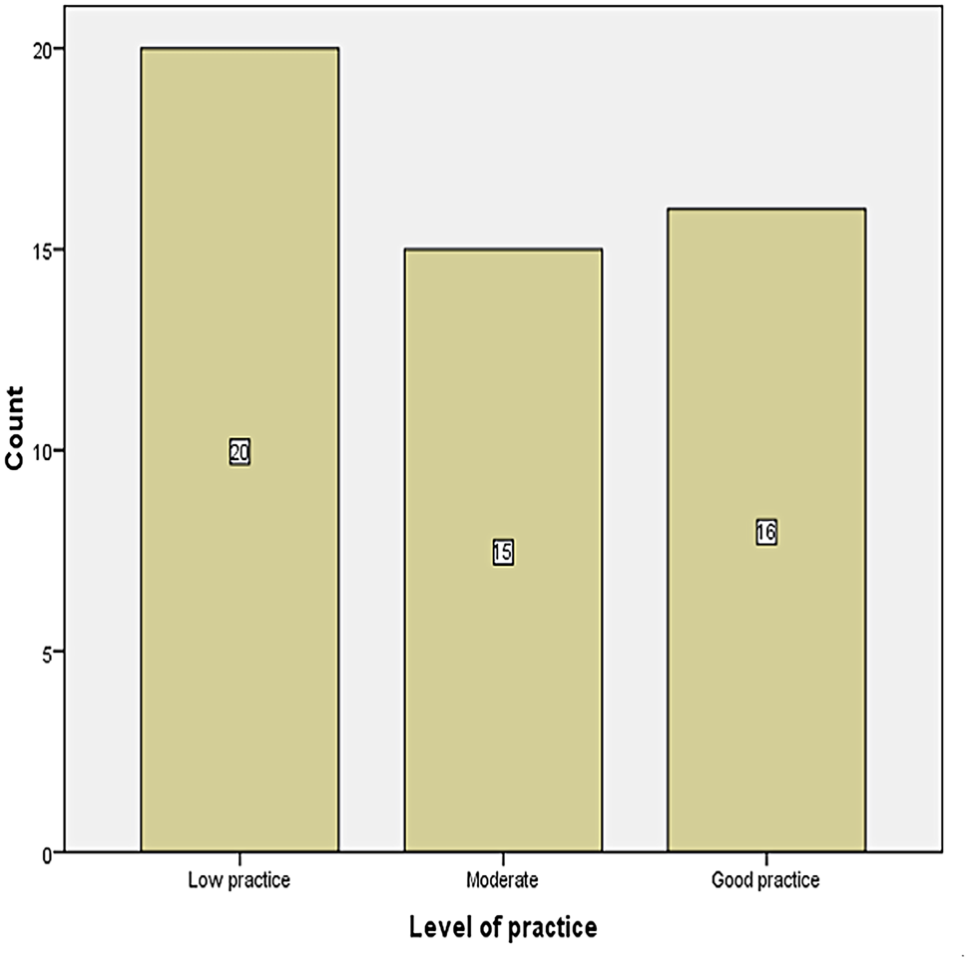
Level of practice towards management of sepsis among nurses working in ICU/HDU

Figure 2 illustrates the distribution of nurses’ level of practice in managing sepsis, with a notable proportion demonstrating low practice. The level of practice was assessed, among them 20(39.2%) showed low practice, 16(31.4%) good practice.

This Table 4 presents the ordinal logistic regression analysis identifying factors associated with nurses’ knowledge of sepsis management.

Nurses with a Bachelor’s Degree in Nursing had significantly lower knowledge levels compared to those with a Master’s Degree (Estimate = -2.999, *p* = 0.032, CI: -5.738 to -0.260).

**Table 4:** Ordinal logistic regression of factors associates with knowledge

Variables		Estimate	Std. Error	Sig.	95% Confidence Interval	
					Lower Bound	Upper Bound
Threshold	[score_cat = 1.00]	-3.648	3.454	0.291	-10.418	3.123
	[score_cat = 2.00]	− 0.036	3.446	0.992	-6.791	6.719
Location	[Gender = Female]	-1.105	0.880	0.209	-2.830	0.620
	[Gender = Male]	0^a^	.	.	.	.
	[age = 20–29]	− 0.234	1.730	0.892	-3.625	3.157
	[age = 30–39]	0.649	1.389	0.640	-2.074	3.371
	[age = 40–49]	0.505	1.549	0.744	-2.531	3.542
	[age = 50 and a]	0^a^	.	.	.	.
	[marital = Married]	0.102	2.360	0.965	-4.522	4.727
	[marital = Single]	-1.054	2.469	0.669	-5.894	3.785
	[marital = widow]	0^a^	.	.	.	.
	[Level = Bachelor’s Degree in Nursing]	-2.999	1.398	**0.032**	-5.738	− 0.260
	[Level = Diploma in Nursing]	-2.035	1.357	0.134	-4.696	0.626
	[Level = Master’s Degree in Nursing]	0^a^	.	.	.	.
	[experience = 1–3 year]	0.975	0.994	0.327	− 0.973	2.922
	[experience = 4–6 year]	1.265	1.322	0.339	-1.326	3.856
	[experience = 7 years]	1.402	1.137	0.217	− 0.826	3.631
	[experience = Less than 1 year ]	0^a^	.	.	.	.
	[trained = No]	0.638	0.789	0.418	− 0.908	2.185
	[trained = Yes]	0^a^	.	.	.	.

- Other variables such as gender, age, marital status, work experience, and training were not significantly associated with knowledge (p-values all > 0.05)

- The model suggests that educational level, particularly having only a bachelor’s degree, is a strong predictor of limited knowledge on sepsis (Table 4)

The results of the ordinal logistic regression revealed several important predictors of nurses’ practice levels in sepsis management. Notably, training in sepsis management was significantly associated with better practice outcomes. Nurses who had not received training were more likely to demonstrate inadequate practices, with a statistically significant association (*p* = 0.002; Estimate = -3.302; 95% CI: -5.404 to -1.199).

Furthermore, educational attainment was also a significant factor. Nurses holding a Bachelor’s degree in Nursing had significantly lower odds of inadequate practice compared to those with a Master’s degree (*p* = 0.021; Estimate = -3.279; 95% CI: -6.061 to -0.498). This suggests that higher academic preparation may positively influence competency in sepsis care. However, no statistically significant associations were found between practice and variables such as gender, age, marital status, or years of experience (*p* > 0.05 across categories).

These findings underscore the importance of both formal education and specific training in improving clinical practices related to sepsis management (Table 5).

**Table 5:** Ordinal logistic regression of factors associates with practice

Variables		Estimate	Std. Error	Sig.	95% Confidence Interval	
					Lower Bound	Upper Bound
Threshold	[prac_cat = 1.00]	-8.670	3.783	0.022	-16.085	-1.256
	[prac_cat = 2.00]	-6.771	3.688	0.066	-14.000	0.458
Location	[Gender = Female]	-1.114	0.962	0.247	-2.999	0.771
	[Gender = Male]	0^a^	.	.	.	.
	[age = 20–29]	-2.280	1.891	0.228	-5.986	1.427
	[age = 30–39]	0.198	1.509	0.896	-2.760	3.156
	[age = 40–49]	0.775	1.631	0.635	-2.422	3.971
	[age = 50 and a]	0^a^	.	.	.	.
	[marital = Married]	-2.576	2.117	0.224	-6.726	1.574
	[marital = Single]	-2.951	2.252	0.190	-7.366	1.463
	[marital = widow]	0^a^	.	.	.	.
	[Level = Bachelor’s Degree in Nursing]	-3.279	1.419	**0.021**	-6.061	− 0.498
	[Level = Diploma in Nursing]	-1.318	1.279	0.303	-3.826	1.189
	[Level = Master’s Degree in Nursing]	0^a^	.	.	.	.
	[experience = 1–3 year]	− 0.223	0.965	0.817	-2.114	1.668
	[experience = 4–6 year]	-1.462	1.287	0.256	-3.985	1.061
	[experience = 7 years]	1.225	1.105	0.268	− 0.941	3.390
	[experience = Less than 1 year ]	0^a^	.	.	.	.
	[trained = No]	-3.302	1.073	**0.002**	-5.404	-1.199
	[trained = Yes]	0^a^	.	.	.	.

## Discussion

This cross-sectional study was conducted at Rwanda’s national referral hospital to assess nurses’ knowledge and practice in sepsis management. While literature suggests that experience enhances knowledge, this study found no significant association between years of experience and sepsis knowledge. A study carried out in the Emergency Department of Aga Khan University Hospital, Karachi, Pakistan, between August and October 2017. A total of 53 healthcare providers participated. Overall, 42 participants (79%) demonstrated a good knowledge of the sepsis. The most commonly reported barrier to compliance with the bundle was staff shortage (62%), followed by delayed patient presentation (58%) and overcrowding in the emergency department (42%), based on our findings at CHUK, only 29.4% of nurses had good knowledge of sepsis, This shows a notable difference in baseline knowledge levels [15].This disparity may result from the difference between healthcare systems and the availability of various nursing specialties.

A study conducted in Mulago National Referral Hospital found that over 70% of ICU nurses had good knowledge and practice regarding sepsis management. This contrasts with our study where only 29.4% had good knowledge and 39.2% showed poor practice. The Ugandan study attributed better outcomes to regular in-service training and established sepsis protocols [12]. The results from current study showed that only 11.7% had low level of knowledge. The results from our study are different from the results of the study conducted in Jordan where 82.9% had inadequate knowledge towards sepsis management [16].

The results of the current study are also different from the results of the study conducted in Ethiopia, where they found that, participants with low level of knowledge were 56.7% [17]. The results of the study conducted in Jordan are higher than the results of the current stud [18] in their study, they found that 50% reported having adequate knowledge caring for patients with sepsis. Another study conducted in china showed that nurses had moderate knowledge, good attitudes and good practices regarding diagnosing and managing of sepsis [19].

The disparity may result from small sample size, which comprised just 51 participants. In addition, the current finding is inferior to the Egyptian study [19].

68% of participants knew too little about managing sepsis. The causes of variations could be study population, sample size and study area. In our study, this moderate level of knowledge was corresponding with low level of practice towards sepsis. The findings of the current study was supported by the results of previous studies from which poor knowledge and practice towards sepsis were reported [17, 20].

The moderate knowledge and poor practice of sepsis management reported in the present study may be caused by different factors including Rwandan nursing schools’ curriculum, which do not cover sepsis content adequately. Another factor may be the lack of proper continuous training and staff development seminars on sepsis management for nurses. Contrary to other studies that suggest that gaining a high degree can help one’s knowledge and practice base grow and become proficient, the current study revealed that bachelor’s degree holding nurses showed low level of knowledge towards sepsis.

The low level of practice was reported in our study; this low level of practice was expected since 21% of participants said they have received specific training in sepsis management. According to Abd-Allah [21], Keeping nurses’ knowledge and practice up to date through ongoing in-service training programs that highlight the significance of the most recent evidence based practice regarding sepsis in continuing education and training programs are necessary, as is the provision of regular training sessions for currently hired intensive care unit nurses on the topic Trained nurses are more accustomed to providing care and applying their skills for patients with sepsis. This is supported by the study conducted by Teshager W.,Getachew B. and Habtamu Getachew in Ethiapia [22], the study revealed that participants who had received in-service training on infection prevention were more likely to exhibit good practices. The results from the present study, are supported by the results from the study conducted in Ethiopia by Alaro et al. [18], respondents who had not received formal training on sepsis management were 2.5 times more likely to have poor knowledge compared to those who received such training. This is supported by different literature [23, 24] indicating that providing formal training towards sepsis management could have a positive relation with increasing nurses’ knowledge. This could be because training on the most recent sepsis management recommendations will increase understanding of sepsis management, which significantly contributes to the delivery of high quality of care.

Globally, the findings resonate with other studies in low- and middle-income countries. Korhan et al. in Turkey reported that while nurses had a general awareness of sepsis, their practical skills were limited due to a lack of structured training and experience [25]. Similarly, Alenezi et al. in Saudi Arabia found nurses demonstrated moderate knowledge but lacked confidence in clinical application, largely due to insufficient training [26]. Our study’s finding that training is a significant predictor of practice quality (*P* = 0.002) strongly reinforces these observations. In direct contrast, high-income countries like the UK show different outcomes; Nugent et al. observed improved nurse competence attributed to regular guideline implementation and robust institutional training [27]. This disparity underscores the critical role of systemic support and consistent training programs, which appear less prevalent in resource-constrained environments like Rwanda.

Educational level emerged as a significant influencing factor. Nurses with only bachelor’s degrees demonstrated significantly lower knowledge than those with master’s degrees (*P* = 0.032). This finding is strongly corroborated by international literature: a study from Australia linked higher educational attainment with better sepsis recognition and more confident intervention [28]. Park et al. in South Korea similarly found that nurses with higher education levels showed significantly better knowledge and decision-making in sepsis care [29], and Ali AA et al. in Egypt noted that educational level significantly determined nurses’ ability to recognize sepsis symptoms and initiate timely interventions [30]. This consistent global evidence highlights higher academic preparation as a pivotal factor for clinical competence in sepsis management.

Regionally, the knowledge-practice gap remains a widespread concern, mirroring our findings. A Nigerian study by Odetola indicated that nurses had limited understanding of sepsis indicators and failed to initiate timely interventions due to knowledge and system barriers [31]. Similarly, Getachew et al. in Ethiopia found that only 34.6% of nurses had good knowledge of sepsis, with most lacking formal training [32]. Tadesse et al., also in Ethiopia, further reported that nurses with advanced academic qualifications had better knowledge of sepsis and adhered more closely to clinical guidelines [33]. These regional studies collectively confirm our results and stress the urgent need for targeted education and training programs across Africa. Again, this contrasts with the more positive outcomes reported in settings with established institutional training frameworks [28]. Research conducted in tertiary hospitals in Cape Town found no significant correlation between nurses’ educational level and their knowledge or practice regarding sepsis. This contrasts with our study, which identified educational attainment as a significant predictor of both knowledge and practice [34]. A study conducted in a tertiary hospital in India found that years of ICU experience were positively associated with better sepsis knowledge and faster response times. In contrast, our study at CHUK found no significant relationship between work experience and nurses’ knowledge levels [35].

Locally, limited prior research directly explored sepsis competencies, but existing evidence aligns with our study. Mugeni et al. examined emergency nursing knowledge in Rwanda and identified a general lack of readiness among nurses to manage critical conditions, often due to training and resource limitations [36]. Our study supports this, adding specific evidence related to sepsis and underscoring the necessity of enhancing training opportunities in tertiary hospitals like CHUK.

Finally, consistent with multiple international studies, demographic factors such as age, gender, marital status, and years of experience showed no significant association with either knowledge or practice in our study. This suggests that focused education and targeted training are more critical determinants of clinical competence in sepsis management than demographics alone.

### Limitations and strength of the study

The study used a cross-sectional design limits the ability to establish causal relationships between the identified factors (such as education or training) and nurses’ knowledge or practice. This design captures data at a single point in time, which does not account for changes in knowledge, behavior, or system-level interventions over time. Longitudinal studies are therefore recommended for a more comprehensive understanding of how training and experience impact sepsis care. The use of self-administered questionnaires may have introduced response bias, potentially leading to overestimation of skill. Conducting the study at a single tertiary hospital also limits the generalizability of the findings to other settings. Additionally, the study did not explore nurses’ personal experiences or contextual barriers in managing sepsis, which qualitative methods could address.

Despite these limitations, the study offers valuable baseline data for future researchers regarding nurses’ knowledge and practice about sepsis management in Rwanda. These findings are critical for informing targeted training and improving care in the ICU/HDU at Kigali University Teaching Hospital.

## Conclusion

This study found that while nurses had moderate knowledge of sepsis, their clinical practice remained insufficient, largely due to gaps in education and lack of specialized training. These results highlight the pressing need for continuous professional development, including regular workshops and structured sepsis-focused training. To enhance sepsis care, nursing leaders and policymakers should implement evidence-based guidelines and support in-service capacity building. Standardizing clinical protocols and reinforcing hands-on skills will be essential to bridge the knowledge-practice gap. Future qualitative research is recommended to explore the real-world challenges nurses encounter in sepsis management and inform more tailored intervention.

## Supplementary Information

Below is the link to the electronic supplementary material.

Supplementary Material 1

## Data Availability

The datasets generated and/or analyzed during the current study are available from the corresponding author upon reasonable request. All relevant materials will be shared in accordance with applicable ethical guidelines and data-sharing policies.

## Abbreviations

CHUK: Centre Hospitalier Universities de Kigali
KUTH: Kigali University Teaching Hospital
ICU: Intensive care unit
HDU: High Dpendency Unit
SIRS: Systemic Inflammatory Response Syndrome
SSC: Surviving Sepsis Campaign
SSI: Surgical site infection
QSOFA: Quick Sequential Organ Failure Assessment
