# Supplementary material for: Evaluation of nurses’ knowledge and practice regarding sepsis management: “A case study of adult ICU/HDU setting at CHUK”
Source: BMC Nurs. 2025 Oct 14;24:1270. doi: 10.1186/s12912-025-03936-7 (PMC12522447; doi:10.1186/s12912-025-03936-7)

**ENGLISH VERSION QUESTIONNAIRE**

**Instrument Development and Validation**

Informed by the prior work of Nakiganda et al. and Manika et al., the questionnaire was initially pretested among nurses in the Pediatric Intensive Care Unit (PICU) to ensure its alignment with the study's objectives. Feedback from this phase guided revisions to improve clarity, relevance, and contextual accuracy. The refined tool was then evaluated by a panel of experts, including seasoned critical care nurses and a senior ICU physician with extensive experience in sepsis management. The final version achieved a Content Validity Index (CVI) of 0.86, indicating strong content validity and suitability for the study(8,9).

**SECTION 1: DEMOGRAPHIC INFORMATION**

**1.Gender:**

a.Male ☐

b.Female ☐

**2.Age:**

a.20-29 ☐

b.30-39 ☐

c.40-49 ☐

d.50 and above

**3.Marital status:**

a. Single☐

b.Married☐

c.widow☐

d.Divorced☐

**4.Highest Level of Education:**

a.Diploma in Nursing ☐

b.Bachelor’s Degree in Nursing ☐

c.Master’s Degree in Nursing ☐

**5.Years of Experience in ICU/HDU:**

a.Less than 1 year ☐

b.1-3 years ☐

c.4-6 years ☐

d.7 years and above ☐

**6.Have you received any specific training in sepsis management?**

a.Yes ☐

b.No ☐

**SECTION 2: KNOWLEDGE ASSESSMENT OF SEPSIS**(‘Tool 1.pdf’, no date)

**Please select the most appropriate answer for each question.**

**1.What is sepsis?**

a.A severe allergic reaction☐

b.A systemic inflammatory response to infection☐

c.A type of cancer☐

**2.Which microorganisms are most commonly responsible for sepsis?**

a.Bacteria ☐

b.Viruses ☐

c.Fungi ☐

d.All of the above ☐

**3. How do underlying conditions such as diabetes, immunosuppression, aseptic wounds, and chronic kidney disease contribute to the development of sepsis in ICU settings?**

a) They increase susceptibility to infections☐

b) They cause direct tissue damage☐

c) They decrease the effectiveness of antibiotics☐

d) They delay the recognition of sepsis☐

**4.According to the Surviving Sepsis Campaign guidelines, what is the recommended time frame for administering antibiotics after sepsis is suspected?**

a.Within 1 hour ☐

b.Within 3 hours ☐

c.Within 6 hours ☐

d.When the physician confirms sepsis ☐

**5. What role do invasive devices play in increasing the risk of sepsis in critically ill patients?**

a) They introduce microorganisms directly into the bloodstream☐

b) They improve the body's immune response☐

c) They prevent bacterial growth☐

d) They enhance tissue healing☐

**6.What is the initial fluid resuscitation recommendation for a patient with septic shock**

a.500ml of normal saline

b.1-2 liters of crystalloids within the first hour

c.No fluids are needed if blood pressure ii stable

**7. Which of the following is a common sign of sepsis? (Select all that apply)**

a.Fever or hypothermia

b.Rapid heart rate

c.Low blood pressure

d.Increased urine output

**SECTION 3: COMPETENCY IN SEPSIS MANAGEMENT**

**1.How often do you assess the risk of sepsis in ICU/HDU patients?**

a.Every shift ☐

b.Daily ☐

c.Weekly ☐

d.Only when sepsis is suspected ☐

**2.Which of the following is the best first-line treatment for sepsis?**

a.Intravenous antibiotics ☐

b.Oral antibiotics ☐

c.Antiviral medications ☐

d.Fluid restriction ☐

**3.What laboratory tests are essential for diagnosing sepsis? (select all that apply)**

a.Blood cultures☐

b,Complete blood count☐

c.Liver function tests☐

d.Urinalysis☐

**4.How familiar are you with the Sepsis Six care bundle (oxygen therapy, blood cultures, antibiotics, fluids, lactate, urine output)?**

a.Very familiar ☐

b.Not familiar ☐

**5.How do hospital-acquired infections, such as ventilator-associated pneumonia or catheter-associated bloodstream infections, contribute to the development of sepsis in ICU settings?**

a) They introduce resistant microorganisms that are difficult to treat☐

b) They reduce the need for infection control measures☐

c) They are rarely associated with sepsis☐

d) They enhance the effectiveness of antibiotics☐

**6.** **How do early identification and timely intervention impact the prognosis of sepsis in ICU patients?**

a) They significantly reduce mortality rates☐

b) They delay the progression of organ failure☐

c) They have no impact on the outcome☐

d) They only reduce the need for antibiotics☐

**7.What are the key factors that contribute to the rapid progression of sepsis in ICU patients?**

a) Delayed diagnosis and treatment☐

b) Lack of invasive monitoring☐

c) Increased physical activity☐

d) Overuse of prophylactic antibiotics☐

**8. What resources do you utilize for information on sepsis management? (Select all that apply)**

a.Clinical guidelines☐

b.Online courses☐

c.Workshops/conferences☐

d.Peer discussions☐

**9. How do organ dysfunction and sepsis-induced immunosuppression interact in critically ill ICU patients?**

a) Organ dysfunction worsens sepsis-induced immunosuppression☐

b) Organ dysfunction reduces the severity of sepsis☐

c) Immunosuppression helps protect organs from damage☐

d) They have no significant interaction☐

**10. Have you participated in any training or educational programs focused on sepsis management in the past year?**

Yes☐

No☐

**Thank you for your collaboration and contribution in this research.**

**APPROVAL FROM CHUK ETHICS COMMITTEE.**


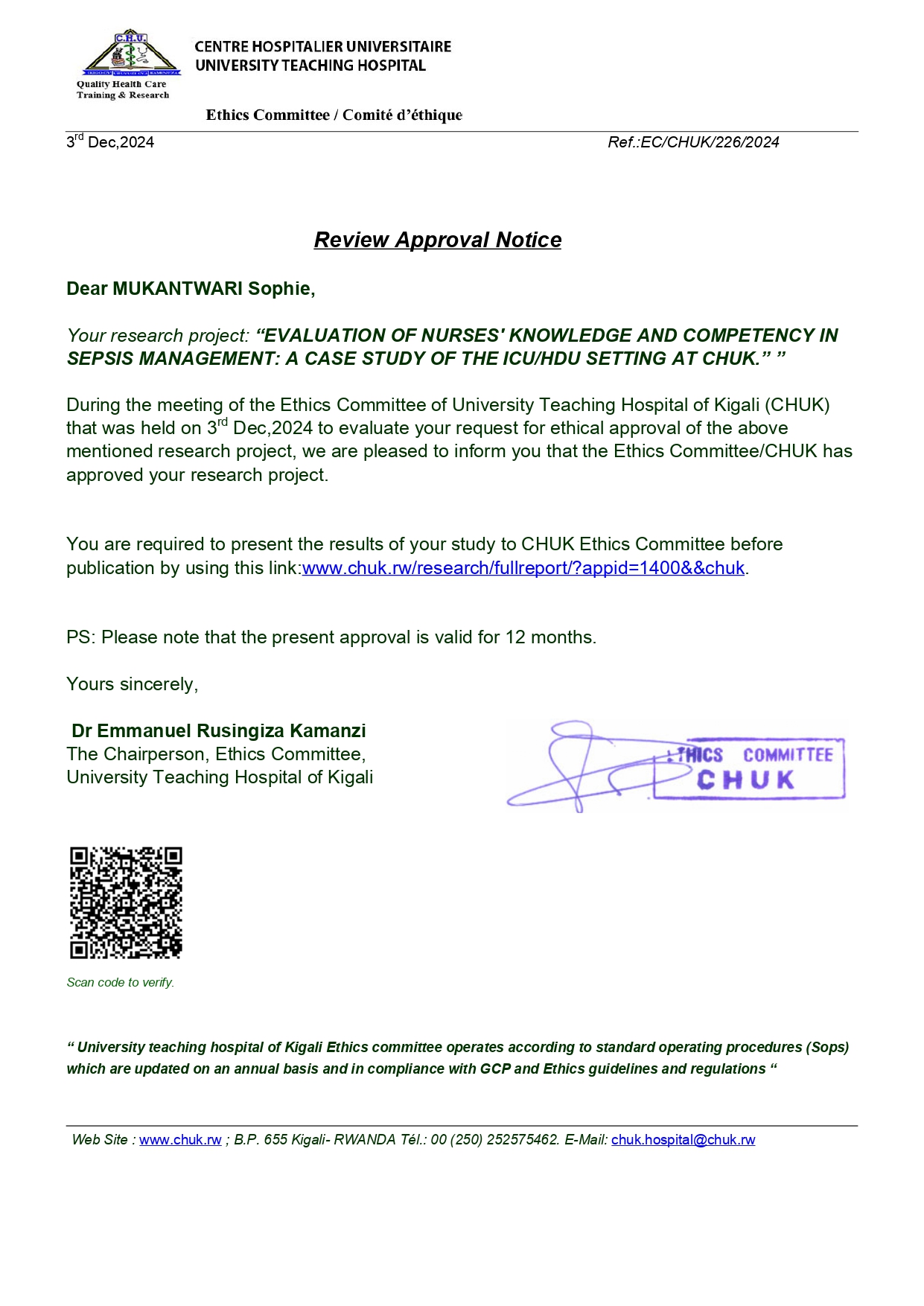

Supplement: Supplementary file 1 — Supplementary Material 1 [file 12912_2025_3936_MOESM1_ESM.docx]
